# The Role of Chemokine Receptor CXCR3 and Its Ligands in Renal Cell Carcinoma

**DOI:** 10.3390/ijms21228582

**Published:** 2020-11-14

**Authors:** Monika Gudowska-Sawczuk, Jacek Kudelski, Barbara Mroczko

**Affiliations:** 1 Department of Biochemical Diagnostics, Medical University of Bialystok, Waszyngtona 15A St., 15-269 Bialystok, Poland; mroczko@umb.edu.pl; 2 Department of Urology, Medical University of Bialystok, M. Skłodowskiej-Curie 24A St., 15-276 Bialystok, Poland; jkudelski@op.pl; 3 Department of Neurodegeneration Diagnostics, Medical University of Bialystok, Waszyngtona 15A St., 15-269 Bialystok, Poland

**Keywords:** CXCR3, chemokines, renal cell carcinoma, metastasis

## Abstract

The major invasive subtype of kidney cancer is renal cell carcinoma (RCC). The essential components of cancer development are chronic inflammation and neoangiogenesis. It has been suggested that the chemokine ligand 9, -10, –11 (CXCL9–11) and chemokine receptor 3 (CXCR3) chemokines receptor expressed on monocytes, T and NK cells may be involved in the inhibition of angiogenesis. However, to date, little is known about the potential clinical significance of these chemokines and their receptor in renal cell carcinoma. Therefore, in this review, we described the role of CXCR3 and its ligands in pathogenesis of RCC. We performed an extensive search of the current literature in our investigation, using the MEDLINE/PubMed database. The changes of chemokines and their specific receptor in renal cell carcinoma were observed. Published studies revealed an increased expression of CXCR3 and elevated concentration of its ligands in RCC. The association between treatment of RCC and CXCL9–11/CXCR3 concentration and expression was also observed. Moreover, CXCR3 and its ligands levels were related to patient’s prognosis, risk of metastasis and tumor growth. This review describes the potential role of CXCR3 and its ligands in pathogenesis of RCC, as well as their potential immune-therapeutic significance. However, future studies should aim to confirm the clinical and prognostic role of CXCL9–11/CXCR3 in renal cell carcinoma.

## 1. Introduction

Kidney cancer is the 13th most common malignancy in the world. It is estimated that more than 300,000 new cases are diagnosed yearly. The highest incidence rates of kidney cancer are observed in Northern Europe, Northern America, New Zealand and Japan. To date, the cause of kidney cancer is unknown. Various risk factors are suggested to play an important role in the overall development of renal cell cancer, i.e., obesity, smoking and hypertension [1]. However, it is well known that the risk actually increases with age of a patient and that the prevalence of kidney cancer is two times more frequently in men in comparison to women [2,3,4]. The most common and the major invasive subtype of kidney cancer is renal cell carcinoma (RCC). RCC, although being rare, represents up to 3% of all malignant visceral neoplasms, thus, it is the insidious cancer [5]. It develops asymptomatically for a long time and makes its presence known when the disease is advanced, and metastasis occurs. From what is currently known, 40% of patients with RCC die, because it is the most lethal urological tumor [5]. Approximately one-third of patients present with metastatic cancer [6]. Histologically, RCC is classified into subtypes. The very common RCC type is clear cell carcinoma, with a frequency up to 75–80% [4,5]. In addition, RCC can be divided into papillary, chromophobe and collecting duct types or a very rare RCC group which includes, e.g., carcinoma of the collecting ducts of Bellini and renal medullary carcinoma. However, independently of the RCC subtype, this cancer is often associated with poor prognosis for patient because of late diagnosis [5,7]. Therefore, accurate and fast diagnosis is particularly crucial for cancers of the kidney. When cancer is detected in its early stages, patients have more treatment options and a greater chance of survival [7].

Imaging techniques (ultrasound, magnetic resonance imaging, computed tomography) play a crucial role in the diagnosis and staging of patients with RCC. In addition, biopsy, which allows for an examination of tissue or cells, is the current method used for the final diagnosis of RCC [8,9]. However, if any pathology in urinary system is suspected, the fundamental diagnostic is based on urine tests and morphology. The application of biochemical blood tests in the diagnosis and management of RCC is also well known. Several potential biochemical serum diagnostic biomarkers might be used in the diagnosis of RCC patients, such as vascular endothelial growth factor (VEGF) or serum amyloid A (SAA) [10]. Moreover, it has been proven that antiapoptotic proteins or the inhibition of proapoptotic mediators have an abnormal activity in RCC and, during apoptosis, death renal cells release cellular contents, e.g., the tumor necrosis factor-related apoptosis-inducing ligand (TRAIL) [11,12].

Currently, we can distinguish two types of the tumor microenvironment: “cold” and “hot”. The first is associated with cytokines i.a. IL-4 and IL-10. The second is connected with a high density of CD8+, CD4+ tumor-infiltrating lymphocytes, monocytes and IFN stimulated chemokines [13]. Numerous studies have been done regarding the ability of CXC chemokines to promote antitumor activity. Therefore, the aim of this study was to carry out a review of the current literature regarding chemokine receptor 3 (CXCR3) and its ligands, to determine their clinical significance and role in the pathogenesis of renal cell carcinoma.

## 2. Methods

**Figure 1:**
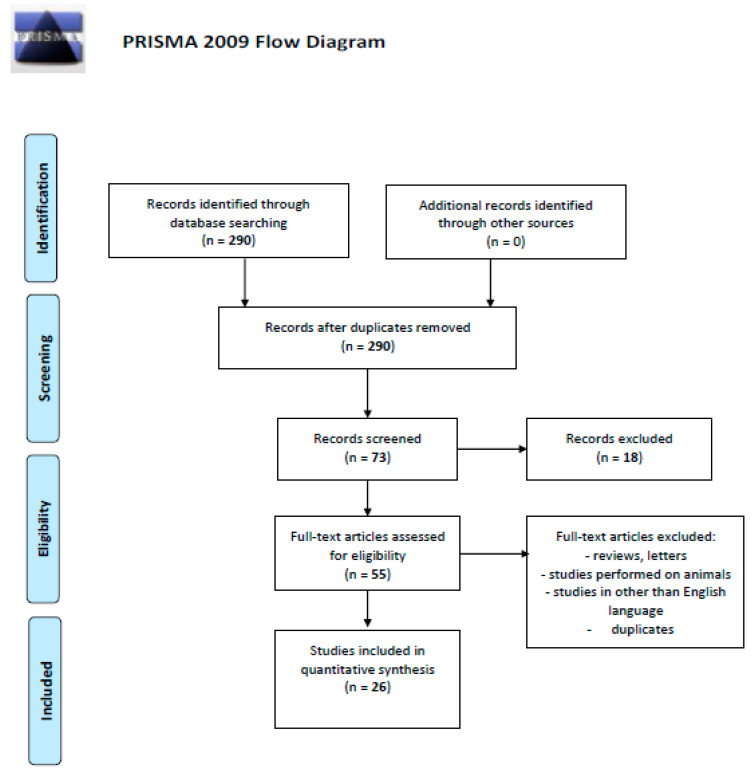
Schematic illustration of articles included in the review manuscript [14].

We performed a comprehensive literature search covering the period up to June 2020 using the MEDLINE/PubMed electronic database with the following search strategy: keywords, “chemokines AND renal AND cell AND carcinoma” (290 studies). When we used the keywords “CXCR3 AND renal AND cell AND carcinoma”, a total of 27 papers were found. A search including the keywords “CXCL9 AND renal AND cell AND carcinoma” produced a total of 13, “CXCL10 AND renal AND cell AND carcinoma”—21 and “CXCL11 AND renal AND cell AND carcinoma”—12 papers. The next step involved limiting the search to human studies written in English. Following this, the search was narrowed down to research studies published within the last 20 years. In the final step, we excluded duplicated papers, all letters to the editor and review papers. Thus, 26 original publications on CXCR3 and chemokine ligand 9–10 (CXCL9–10) in renal cell carcinoma were included in the study (Figure 1. Schematic illustration of articles included in the review manuscript [14]).

## 3. Results and Discussion

Chemokines are a group of chemoattractant soluble cytokines involved in inflammation. This proteins family is a key regulator of tumor angiogenesis, which may stimulate T lymphocytes and NK cells movement and localization into the cancer tissue. Chemokines bind and activate specific receptors and receptors respond to chemokine ligands, respectively [15,16,17]. Chemokine receptor 3 also called G protein-coupled receptor 9 (GPR9) and CD183 has three different variants: CXCR3-A, CXCR3-B and chemokine receptor 3-alternative (CXCR3-alt) [18,19,20]. CXCR3 exerts its biological effect by binding to three interferon γ (INF-γ; Typẹ ỊI interferon)-inducible ligands: chemokine ligand 9 (CXCL9), chemokine ligand 10 (CXCL10) and chemokine ligand 11 (CXCL11) [21]. It has been proven that the CXCR3 expression correlates with CD4+ Type-1 helper (Th1) and CD8+ cytotoxic lymphocytes, and that chemokines CXCL9–11 are greatly elevated in patients with renal cell carcinoma in comparison to healthy ones. In addition, the expression of human CXCR3 related chemokines is associated with tumor development, and the alteration of these chemokines expression is probably related to prognosis in patients with RCC. CXCL9 and CXCL10 exhibit antitumor activity, which has been proven e.g., in studies on mice. CXCL10 is also responsible for reducing the levels of VEGF, fibroblast growth factor and matrix metalloproteinase-9. Higher levels of chemokines bind to CXCR3 in the RCC patients are closely associated with their angiostatic function [22,23,24,25,26]. The significance of CXCL9–11/CXCR3 in renal cell carcinoma is presented in Table 1.

Standard renal cell carcinoma treatment includes surgery, targeted therapy and immunotherapy. These treatments can be used alone or combined with each other. However, conventional chemotherapy and radiation in advanced RCC is essentially ineffective [45]. Immunotherapy is a promising, but complicated strategy of treatment. RCC immunotherapy comes in a variety of forms, and it is well known that CD8+ and CD4+ cells play crucial role in immunotherapy, because of the ability of independent antitumor action i.a., the recognition of antigenic epitopes on tumor cells or control and modulation of the host immune response during tumor development. These lymphocytes are responsible for the recognition of abnormal cancer cells that are rapidly eliminated by triggering cytotoxicity mechanisms [46,47,48]. According to the function, T lymphocytes can be subdivided into various types of i.a., helper cells (Th). Since Th1 cells stimulate the production of the IFN-γ and mediate cellular immune response, whereas Th2 cells induce humoral immunity [49]. It has been proven that chemokine receptors are located on the surface of T cells, and their condition depends on stimulation with antigens [50]. As a result of chemokine ligand binding, the chemokine receptors promote many signaling cascades, which affect a cellular response. Importantly, it has been described that in patients with the tumor, the levels of Th1-related chemokines and INF-γ are elevated, in comparison to normal kidney tissue [24]. It has been proven that CXCL9–11 are Th1-associated chemokines. An elevated secretion of chemokines induces the recruitment of T cells and natural killer (NK) cells into the cancer tissue to stimulate antitumorigenic activities [24,27,28]. Due to the ability of increasing the immune response, many chemokine receptors and adhesion molecules have been studied by Oldham et al. It has been found that CXCR3, CC chemokine receptor 5 and CXC chemokine receptor type 6 are over expressed on tumor-infiltrating lymphocytes (TILs). Hence, this suggests that CXCR3 located on Th-1 cells are associated with inflammatory type 1 Th response while CXCL9–11 are regularly found at the site of inflammation. Therefore, it is important to mention that CXCR3 ligands in RCC tissue correlates with enlarged T-cell infiltration [36,37]. In addition, regression of RCC is related to Th1 lymphocytes activity [24]. The subpopulation of T lymphocytes, regulatory T cells (Tregs) are also essential in the regulation of immune system. These cells are involved in production of immunosuppressive cytokines and some chemokines, leading to the attraction of CD4+ and CD8+ cells in local tissues [51]. The role of T cells and chemokines in RCC as markers of immunity has been rarely investigated in the *Polimeno* et al. study. This study evaluated profile of T cells, NK cells and cytokines/chemokines in RCC. Authors observed an elevated levels of Treg CD4+ in those patients. Additionally, a markedly higher levels of the CXCL10, CXCL11 and other molecules e.g., IL-4, IL-6, VEGF in peripheral blood were observed. High concentrations of these two chemokines were significantly higher in post-nephrectomy RCC-free patients in comparison to healthy patients. Additionally, CXCL10 correlated with a network associated with movement and proliferation of renal cells. Based on these findings, the authors strongly suggest that CXCL10 and CXCL11 may be surrogate indicators of host immunity in renal cell carcinoma patients [29]. Moreover, the researchers suggest that CXCR3-targeted therapy could potentially be a new useful strategy for the treatment of RCC patients. It may be explained by the fact that suppression of genes encoding CXCR3 ligands is significantly increased at the tumor site, and it known that these chemokines guide the trafficking behavior of T lymphocytes [49].

The effects of clinical drug treatments on CXCR3/CXCR3 ligands expression in renal cell carcinoma was evaluated by treating patients. The expression of the receptor and ligands on the surface of peripheral blood mononuclear cells (PBMCs) and the concentration in serum/plasma before and after immunotherapy may be a useful biomarker predicting tumor response to treatment.

In 2015 nivolumab which is a human IgG4 programmed cell death 1 (PD-1) immune checkpoint inhibitor was one of the first medicaments approved for metastatic RCC, because of its ability to selectively blocking the interaction between PD-1 and its ligands [30]. Choueiri TK et al. aimed to investigate the immunomodulatory activity of Nivolumab on i.a. chemokines [52]. They found a trend towards increased concentrations of serum CXCL9 and CXCL10 as markers of T-cell activation and migration after therapy [52]. After treatment, CXCL9 and CXCL10 levels showed a significant difference between the baseline and the cycle 2 day 8 of treatment. Additionally, median percent changes from baseline of these chemokines were higher for CXCL9 than CXCL10. However, such changes were not associated with the drug dose. As the authors suggest, serum chemokine changes from baseline may reflect nivolumab pharmacodynamics in the RCC microenvironment [52].

These results are in line with the findings presented by *Reckamp* et al. and *Pan J.* who hypothesized that interleukin-2 (IL-2) therapy of metastatic clear cell RCC could increase CXCR3 levels [31,53]. Authors reported augment expression of CXCR3 in PBMCs (CD4, CD8, NK) in response to high dose IL-2 treatment [31,53]. It is worth mentioning that CXCR3 expression was the highest in patients with a complete response to therapy, suggesting CXCR3 may be potentially a significant marker for response to IL-2 therapy [31]. Studies in mice have shown that IL-2 may also led to an elevation of plasma concentration of CXCL-9 and CXCL-10. In parallel, the chemotactic gradient is inhibited that conduces recruitment of CXCR3 mononuclear cells into the cancer tissue. However, in tumor tissue IL-2 treatment caused predominantly elevation of CXCL-9 only. Going forward, scientists observed that the combination of IL-2 with an intratumor CXCL-9 leads to better suppression of cancer cells proliferation and angiogenesis than IL-9 and CXCL-9 alone [53]. Moreover, the angiogenic ratio value calculated using the levels of proangiogenic factors (e.g., CXCL3, VEGF and antiangiogenic ligands of CXCR3 (CXCL9, -10, -11) was elevated before the treatment in RCC patients in comparison to healthy controls. On the contrary, after high dose of IL-2 they observed the angiogenic ratio shifted in favor of the antiangiogenic factors [31].

Histone deacetylases are a group of key regulators of gene expression that are dysregulated in RCC, leading to excessive cell proliferation and differentiation. Therefore, the use of histone deacetylase inhibitors as one of therapy types for RCC. [32,38,39]. Juengel et al., in an experimental study, tried to evaluate the effect of histone deacetylase-inhibitor valproic acid in combination with interferon-α on gene expression of the renal cell carcinoma cells. Their microarray analysis revealed that this combination leads to alterations of already known genes, but also demonstrated an expression of new involved in tumor progression. This study revealed that, after treatment, genes of chemokines associated with CXCR3 are also upregulated. A 79-fold of CXCL10 and 89-fold elevation of CXCL11, in comparison to the control group, was also observed. The microarray data was in accordance with RT qPCR analysis which indicates that CXCR3 ligands are responsible for T lymphocytes and natural killer cells chemoattraction, leading to inhibition of angiogenesis. However, that elevation was inconstant and returns to baseline at 3–5 days. Therefore, the authors suggest that prolonged treatment significantly inhibits RCC proliferation and gives a better outcome than short application of medicaments [38].

Last year Xu W. et al. evaluated the effect of anti-angiogenic VEGFR TKIs on the concentration of plasma chemokines. The authors observed that out of three CXCR3 ligands only CXCL10 was elevated after 4 and 6 weeks of treatment, compared to mean baseline. They also found that patients with increased CXCL10 before therapy showed significantly worse outcomes of RCC in comparison to patients with lower level of CXCL10 [40]. Another study evaluated effects of promising anticancer therapy, cyclophosphamide (CP) on anti-CTL-associated protein 4 (anti-CTLA-4) in the non/low RENCA renal carcinoma mouse model. It turned out that the immune checkpoint blockade therapy is commonly associated with immune related adverse events. Using the Proteome Profiler Mouse Cytokine Array, it was observed that the serum concentrations of type I IFN-induced chemokines, including CXCL10 were elevated in CP-treated models followed by anti-CTLA-4 therapy. Nevertheless, the highest levels of CXCL10 were noted in RENCA mice models treated with CP and anti-CTLA-4 simultaneously [54,55].

It has been suggested that increased concentration of CXCL9 and CXCL10 is a good prognostic factor for patients with RCC. This hypothesis is based on the fact that these CXCR3 ligands may contribute to antitumor defenses inhibition of angiogenesis [23,24]. Furthermore, it is known that cancers develop due to the accumulation of genetic alterations, such as mutation of chromosome 9p is associated with increased renal cancer risk [33,41]. A growing body of evidence indicates that deletion of 4q and 4p also contributes to CD8+ lymphocytes and NK cells exclusion. Xiong et al. evaluated the prognostic landscape of arm somatic copy number alterations and its connection with renal cell carcinoma. They reported that mutations of chromosome 1p, 3p, 4p, 4q, 5p, 5q, 11p, 11q, 13 q and 19p are the risk factors for overall survival and recurrence free survival. However, only 4q deletion leads to downregulation of ligands associated with CXCR3, because the genes of CXCL9–11 chemokines are located on human chromosome 4q. Contrary to Xu et al., the authors suggest that the high risk of death in RCC patients is associated with decreased level of these chemokines and mutation of chromosome 4q [32,38,42,56]. Interestingly, it was also observed that high expression of CXCL10 involved in immune system activation correlate with favorable survival rate in those patients [57]. However, there is something of a discrepancy between prognosis of RCC patients and scientific findings. In 1999 and 2005, the first studies suggesting the role of CXCR3 in cancer metastasis were published. Scientists revealed that expression of CXCR3 is markedly elevated in human renal cell carcinoma in comparison to a normal kidney tissue [25,43]. Liu W. et al. tried to investigate whether elevated expression of CXCR3 has an impact on recurrence and survival of non-metastatic RCC. According to the authors, the serum levels of CXCL9 and CXCL10 are strongly associated with Fuhrman grade and grade and necrosis score (SSIGN). Furthermore, CXCL10 concentration was related with pT stage, necrosis and Mayo Clinic stage. Additionally, this study has shown that CXCL-9, -10 and -11 overexpression is associated with a worse prognosis in RCC. Thus, authors developed a prognostic score based on these values of these ligands and they observed that high IFN-inducible CXCR3 ligands (ICL) score correlate with Fuhrman grade, necrosis and high-risk of SSIGN. Additionally, increasing ICL value was more precise and useful than single chemokine determination and allows stratified patients into subgroups of overall survival and recurrence. This finding may confirm that CXCR3 ligands have a potential role in stimulating tumor growth and spread via an autocrine manner [44]. Subsequently, based on the knowledge that CXCR3-A induces i.a. human microvascular endothelial cell line-1 migration and proliferation, whereas CXCR3-B stimulate apoptosis and reduced DNA synthesis. Suyama et al. evaluated the expression of CXCR3, its ligands and variants: CXCR3-A and CXCR3-B. RT-PCR analysis revealed that CXCR3 and its ligands were elevated in RCC. Interestingly, the authors calculated CXCR3-A-to-CXCR3-B ratio. The ratio value was significantly increased (1.5-fold) in RCC in comparison to a normal kidney tissue. Moreover, CXCR3, CXCR3-A and the ratio were significantly increased in metastatic carcinoma versus patients without metastasis. Despite the fact that there are no significant functional differences between ligands of CXCR3, the CXCL10 was most expressed in RCC. The correlation between CXCL10 and CXCR3-A was also observed suggesting a potential role of CXCL10 as a biomarker of tumor metastasis [34,43,58]. It has been proven by Wightman et al., who demonstrated that the expression of CXCR3 and elevated secretion of its ligand–CXCL10 is connected with increased risk of metastasis in RCC. Authors showed that CXCL10/CXCR3 promotes RCC metastasis. They observed that serum CXCL10 expression was higher in high metastatic potential cells (P2M3C) in comparison to low metastatic potential cells (P2M5B). Coexpression of CXCR3 and CXCL10 was associated with poor prognosis more than separate expression of CXCR3 or CXCL10 [35]. Moreover, CD14+ monocytes with low or negative HLA-DR expression are considered to be suppressors of T-cells activation [57,59]. Motoshima et al. found a higher percentage of HLA-DRlow/-monocytes in patients with RCC. Their data show that 75% of the patients had lower expression of HLA-DRlow/-monocytes after surgery. Additionally, they observed that it was associated with CXCL10, but also CXCL2 mRNA expression. These chemokines high expression contrasts with lower percentage of CD14+ HLA-DRlow/-monocytes. This study does not evaluate prognosis; however, we can speculate that the high level of these monocytes is associated with poor prognosis in patients with RCC [57].

Interestingly, another study was done with a group of patients with clear cell RCC and aimed to construct a good prognostic prediction system for this tumor type. The mRNA sequencing data of ccRCC was downloaded from a few database and Cox regression analysis was used for screening prognosis-associated genes. The results from this study confirm a total of 263 overlapped the differentially expressed genes and 161 prognosis‑associated genes. In the next stage of this work authors created prognostic prediction system involving 44 genes including CXCL9 and CXCL10. The most common finding of this study is that a 44-gene set would be helpful in a prediction the prognosis of patients with renal cell carcinoma. However, a potential limitations of the study is a relatively small tested group and a lack of previous research studies on this topic. Therefore, the results still require an experimental validation [26].

Apparently, the reported clinical observations are sometimes contradictory, and make it difficult to use them for therapeutic monitoring and prognosis in RCC. This is likely due to the complexity of the cellular composition of the tumor tissue, its microenvironment and that the tissue microenvironment consists of a population of cellular and non-cellular components. Given the different results presented in publications evaluated both, the concentration and expression of CXCL9–11/CXCR3 in various materials, such as serum plasma and PBMCs as an indirect media of tumor pathophysiology, it is difficult to draw conclusions unequivocally. The detailed analysis of heterogeneous populations of RCC cells using, e.g., immunophenotyping of chemokines receptor profile may be useful in identifying the markers involved in the tumor development. Taking the above into account, we suggest that more studies are needed to confirm the significance of chemokines CXCL9–11 and their receptor in renal cell carcinoma.

## 4. Conclusions

It has been proven that neoangiogenesis and inflammation play an important role in the development and progression of many malignancies, including renal cell carcinoma. CXC chemokines and their receptors have been suggested to modulate the growth, proliferation and differentiation of tumor cells. As a result of the fact that the CXCR3 and its ligands are multifunctional chemokines, a number of original papers evaluated their role in pathogenesis of RCC. In this review, we focused on the changes in expression of CXCR3 and concentration of CXCL9–11 in RCC. The published results demonstrated that RCC is associated with elevated expression of CXCR3 and its ligands in RCC. Moreover, the expression and concentration were significantly higher after treatment in comparison to baseline. However, there is some discrepancy between the studies assessing the correlation of CXCL9–11/CXCR3 and the patient’s prognosis. Therefore, more studies are sorely needed to unequivocally confirm the clinical and prognostic role of above mentioned chemokines and their receptor in renal cell carcinoma.

## Figures and Tables

**Table 1:** The significance of chemokine ligand 9–11 (CXCL9–11)/ chemokine receptor 3 (CXCR3) in renal cell carcinoma.

ROLE	CXCR3	CXCL9	CXCL10	CXCL11	References
Presents angiostatic functions	+	+	+	+	[22,23,24,25,26,27,28]
↑ expression/concentration	+	+	+	+	[23,24,27,28,29,30,31,32]
↑ expression is a good prognostic factor		+	+	+	[24,28,29]
↑ expression associated with poor outcome	+ (and CXCR3-A)		+		[32,33,34,35]
Expression correlates with T-cell infiltration	+	+	+	+	[25,30,36,37]
↑ expression/concentration after treatment	+	+	+	+	[32,33,38,39,40,41,42]
↓ expression associated with deletion of 4q chromosome		+	+	+	[43,44]
